# Veneto Region dementia-related mortality during the COVID-19 pandemic: multiple causes of death and time series analysis

**DOI:** 10.1093/eurpub/ckad005

**Published:** 2023-02-27

**Authors:** Cristina Basso, Claudio Barbiellini Amidei, Veronica Casotto, Elena Schievano, Matilde Dotto, Silvia Tiozzo Netti, Manuel Zorzi, Ugo Fedeli

**Affiliations:** Epidemiological Department, Azienda Zero, Padova, Italy; Epidemiological Department, Azienda Zero, Padova, Italy; Epidemiological Department, Azienda Zero, Padova, Italy; Epidemiological Department, Azienda Zero, Padova, Italy; Epidemiological Department, Azienda Zero, Padova, Italy; Epidemiological Department, Azienda Zero, Padova, Italy; Epidemiological Department, Azienda Zero, Padova, Italy; Epidemiological Department, Azienda Zero, Padova, Italy

## Abstract

**Background:**

Older individuals with dementia have been severely affected by the COVID-19 pandemic. There is a lack of in-depth evaluation of mortality trends using both the underlying cause of death (UCOD) and the multiple causes of death (MCOD) approaches. The objective of this study was to determine the impact of the COVID-19 pandemic on dementia-related deaths considering comorbidities and the place of death.

**Methods:**

This retrospective, population-based study was conducted in Veneto, Italy. All the death certificates of individuals aged ≥65 years issued from 2008 to 2020 were analyzed for dementia-related mortality using age-standardized sex-stratified rates of dementia as UCOD and MCOD. Excess in monthly dementia-related mortality in 2020 was estimated by applying Seasonal Autoregressive Integrated Moving Average (SARIMA) model.

**Results:**

Overall, 70 301 death certificates reported dementia (MCOD proportional mortality: 12.9%), and 37 604 cases identified it as UCOD (proportional mortality: 6.9%). In 2020, the MCOD proportional mortality increased to 14.3% whereas that of UCOD remained static (7.0%). Compared to the SARIMA prediction, MCOD increased by 15.5% in males and 18.3% in females in 2020. Compared to the 2018–19 average, deaths in nursing homes increased by 32% in 2020, at home by 26% and in hospitals by 12%.

**Conclusions:**

An increase in dementia-related mortality during the first months of the COVID-19 pandemic could only be detected using the MCOD approach. MCOD proved to be more robust, and hence, should be included in future analyses. Nursing homes appeared to be the most critical setting which should guide establishing protective measures for similar situations.

## Introduction

Dementia is globally recognized as a leading cause of mortality and its prevalence is likely to increase further because of the increasing aging population.^1^ People with Alzheimer’s disease (AD) and related/other dementias (ADRD) have an increased risk of both severe acute respiratory syndrome coronavirus 2 (SARS-CoV-2) infection and more severe coronavirus disease 2019 (COVID-19).^2^ Older people with ADRD may be unable to follow preventative measures, such as physical distancing, or fail to monitor and report symptoms, thereby increasing the risk of COVID-19 transmission, especially in nursing homes.^3^ Furthermore, patients with dementia are usually older in age and have multiple comorbidities, which are established risk factors for contracting severe COVID-19. In a pooled analysis, July et al.^4^ reported increased dementia-related mortality in patients with COVID-19 and found a clear correlation of mortality with age and comorbidities.

Compared with 2019, patients with ADRD showed a 25.7% increase in overall mortality in 2020, which was twice as much as that observed in individuals without dementia.^5^ Among the COVID-19-associated mortalities in Italian hospitals, 15.8% of the patients had dementia with a frequently atypical clinical presentation and faster deterioration.^6^

The population-based mortality statistics for dementia were heavily influenced by the pandemic. During the first wave (March–April 2020), ADRD-related mortality increased by approximately 14% in Italy.^7^ UK registered a 45% excess ADRD-related mortality in care homes and hospices (March–June 2020).^8^ In the USA, AD-related deaths increased by 9.8%.^9^

These reports are based on routine mortality statistics relying exclusively on the underlying cause of death (UCOD) approach, selected from all the conditions reported on death certificates as per international coding rules. When examining dementia-related mortality, the UCOD approach may be heavily influenced by variations in certification practices and coding rules, as well as the competing causes of death.^10^ During the pre-pandemic period, an increase in ADRD-related deaths was reported in numerous countries, including Australia and the USA; however, this was partly artificial, since it was a consequence of reduced competing causes of death, especially among the older population.^11^

The multiple causes of death (MCOD) approach (i.e. the analysis of all conditions reported in death certificates) is a more robust assessment of mortality trends associated with numerous chronic conditions, including dementia.^11^ This approach is strongly warranted in the current scenario because COVID-19 is likely to be identified as the UCOD in patients with dementia. The limitations of the UCOD approach could explain the reported discrepancies between the overall mortality determined by Gilstrap et al.^5^ and the dementia-related mortality calculated by Ahmad et al.^9^

The present study assessed the impact of the different COVID-19 pandemic waves in 2020 on ADRD-associated deaths in a large population in Northeastern Italy. Comparative data regarding dementia-related mortality obtained from the UCOD and MCOD approaches have been presented and critically discussed considering long-term trends in dementia-related mortality and associated comorbidities, as well as different healthcare settings.

## Methods

The death certificates of all Veneto Region (Northeastern Italy; ∼4.9 million inhabitants) residents are routinely transmitted to the Regional Epidemiology Department and coded according to the International Classification of Diseases, 10th Revision (ICD-10). The standard mortality statistics were based on the UCOD, mostly reflecting the underlying cause assigned by the physician who filled the death certificate; however, other reported diseases or derived conditions might be selected if considered more appropriate. The regional mortality database collects information regarding all diseases listed in the certificate in addition to the UCOD since 2008. Automated Classification of Medical Entities software, a tool developed by the National Center for Health Statistics, has been used to identify the UCOD from 2008 to 2017.^12^ This was replaced in 2018 by a new software, IRIS, which has been adopted in most European countries. The software change slightly changed the rules for selecting the UCOD based on the 2016 version of the ICD-10.

We analyzed the death certificates of the residents of Veneto aged ≥65 years who had dementia (ICD-10 codes: vascular dementia—F01, unspecified dementia—F03 and AD—G30) from 1 January 2008 to 31 December 2020.

Descriptive statistics of the decedents with dementia mentioned on the death certificate were computed and stratified by sex, age and place of death (i.e. at home, in the hospital or in nursing homes). The proportional mortality (share of all deaths) was reported for four different periods: 2008–11, 2012–15, 2016–19 and 2020 (the first year of the pandemic).

The annual age-standardized mortality rates for dementia as the UCOD and MCOD were determined for males and females via direct standardization using the 2013 European population. The average annual percentage change in age-standardized rates (APC) and the relative 95% confidence interval (CI) were calculated for the pre-pandemic period (2008–19) from linear regression models using the logarithm of the age-standardized rates (weighted by the inverse of their variance) as the dependent variable and the corresponding year as the regressor.

The proportion of the most common comorbidities in the study population was investigated for each study period: ischemic heart diseases (I20–I25), cerebrovascular diseases (I60–I69), hypertensive diseases (I10–I13), diabetes (E10–E14), neoplasms (C00–D48), chronic obstructive pulmonary disease (J40–J44, J47) and COVID-19 (U07.1, U07.2). All death certificates mentioning dementia were evaluated according to both the MCOD and UCOD approaches.

The monthly variation in the number of deaths recorded in 2020 compared with their respective 2018–19 average was stratified by place of death and calculated.

To quantify the monthly excess mortality rate of dementia as MCOD in 2020 vs. in 2008–19, a Seasonal Autoregressive Integrated Moving Average model (SARIMA) was adopted.^13^ This method accounted for seasonality and trend, as well as for the correlation between consecutive months and the same month of consecutive years. The SARIMA model was applied to the 2008–19 data to predict the monthly mortality rates in 2020. Excess mortality was estimated as the ratio between the observed rates in 2020 and those expected according to the model.

## Results

Between January 2008 and December 2020, dementia was mentioned in 70 301 death certificates of decedents aged ≥65 years (MCOD approach), with a proportional mortality of 12.9% (table 1). Dementia was listed as the UCOD in 37 604 cases with a proportional mortality of 6.9%. In 2020, the number of reported dementia-related deaths (MCOD) peaked, with over 7400 cases accounting for 14.3% of deaths in the region. At the same time, dementia as the UCOD was reported in 3621 cases with a proportional mortality of 7.0%.

**Table 1:** Dementia-related mortality—underlying (*n* = 37 604) and multiple (*n* = 70 301) causes of death stratified by number, proportional mortality and place of death (2008–20)

Period	2008–11	2012–15	2016–19	2020	Period	2008–11	2012–15	2016–19	2020
**Dementia—underlying cause of death (UCOD)**					**Dementia—multiple causes of death (MCOD)**				
Annual average of deaths (*N*)	2299	2760	3438	3621	Annual average of deaths (*N*)	4498	5203	6023	7406
Male (%)	31.2	30.8	31.4	31.5	Male (%)	31.7	31.4	32.2	33.1
Average age at death (SD)					Average age at death (SD)				
Male	84.2 (6.59)	85.0 (6.60)	85.5 (6.44)	85.7 (6.36)	Male	84.3 (6.50)	84.9 (6.47)	85.5 (6.39)	85.6 (6.32)
Female	87.6 (6.54)	88.3 (6.29)	88.8 (6.18)	88.8 (6.35)	Female	87.6 (6.43)	88.2 (6.16)	88.7 (6.13)	88.8 (6.21)
Age-specific rate × 100 000					Age-specific rate × 100 000				
Male									
65–74 years	26.3	23.5	24.6	23.6	65–74 years	47.8	43.1	43.2	47.9
75–89 years	323.6	320.2	348	340.5	75–89 years	653.4	628.8	631.1	747.2
90 years and over	1785.20	1928.00	2090.90	2057.20	90 years and over	3459.80	3517.50	3713.00	4278.80
Female									
65–74 years	20.4	17.6	19.9	21.2	65–74 years	35.3	30.9	32.4	40
75–89 years	341.8	353.9	385	384.3	75–89 years	672.9	678.7	682.7	780.9
90 years and over	2088.60	2254.30	2539.00	2565.20	90 years and over	4017.30	4109.60	4300.30	5050.90
Proportional mortality (%)					Proportional mortality (%)				
Male	4.1	4.6	5.5	4.8	Male	8.1	8.8	9.9	10.3
Female	9	10.2	12	10.4	Female	14.6	15.7	17.1	17.7
Place of death (%)					Place of death (%)				
At home	n.a.^a^	20.2	19.4	21.1	At home	n.a.	20.7	19.4	19.6
In the hospital	*n.a.*	39.6	38.3	34.2	In the hospital	*n.a.*	42.3	40.6	37.1
In nursing home	*n.a.*	36.9	38.6	42.3	In nursing home	*n.a.*	33.2	36.2	40.5
Other or missing	*n.a.*	3.3	3.6	2.5	Other or missing	*n.a.*	3.7	3.8	2.8

a n.a: data not available.

Overall, females comprised 67.9% of the study population and showed a consistently higher mean age at death compared with males. Age at death only slightly increased in both sexes across the study period, as reported in table 1. Age-specific, sex-stratified mortality rates were slightly higher among males in all the age groups, except for those aged ≥90 years (Supplementary figure a).

The annual age-standardized mortality rates for dementia as MCOD did not vary from 2008 to 2019 in either males (APC: −0.05, 95% CI −0.65 to 0.55) or females (APC: 0.31, 95% CI −0.48 to 1.11). During the same period, dementia as the UCOD increased on average each year by 1.22% in males (APC: 1.22, 95% CI 0.32 to 2.13) and 1.76% in females (APC: 1.76, 95% CI 1.04 to 2.5).

**Figure 1:**
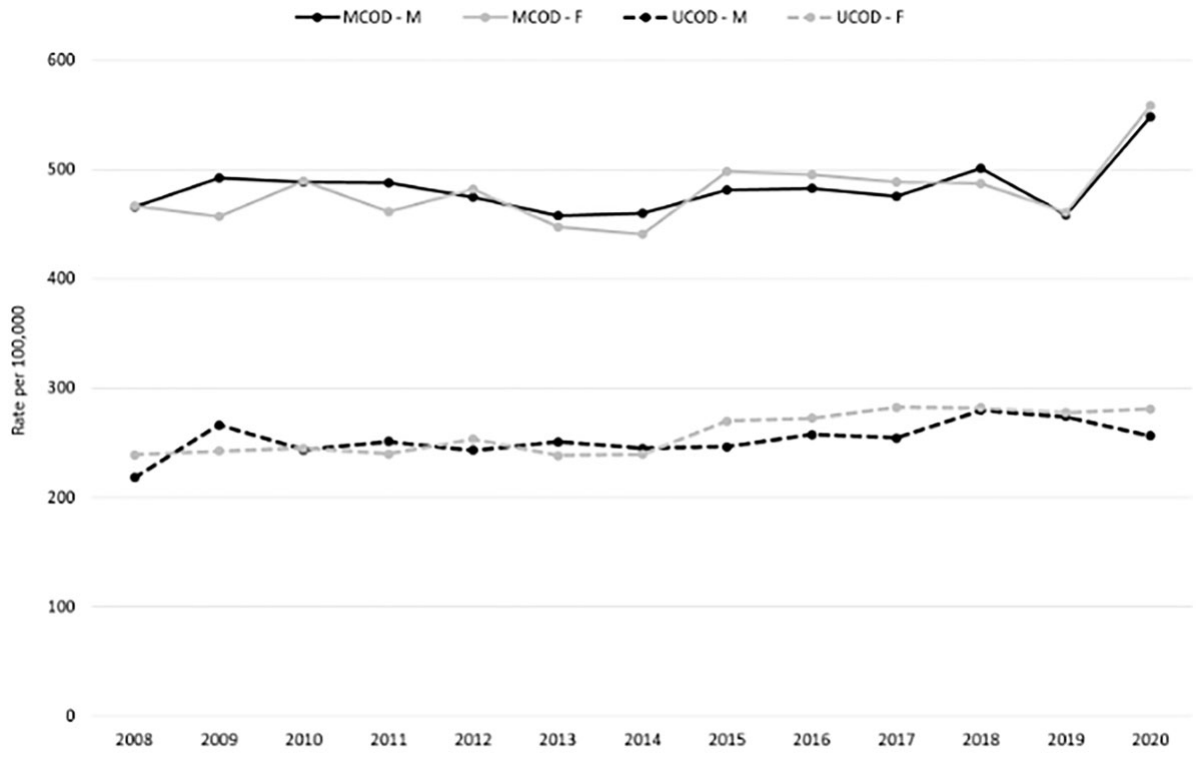
Dementia-related age-standardized* mortality rates (2008–20) by sex using underlying and multiple causes of death approaches. UCOD, underlying cause of death; MCOD, multiple causes of death. *Reference: European population 2013

An increase in age-standardized dementia-related mortality rates was observed in 2020 using the MCOD approach, whereas the UCOD approach exhibited no noteworthy change aside from a slight decrease among males (figure 1 and Supplementary table a).

Dementia was identified as the UCOD in 51.1% and 57.1% of death certificates with the mention of dementia in 2008–11 and 2016–19, respectively (table 2). In 2020, this decreased to 48.9% as a large number of deaths were attributed to COVID-19 (11.8%). Among the most common dementia-related comorbidities, reports describing ischemic heart diseases and cerebrovascular diseases as the UCOD started to decrease from 9.8% and 5.6% in 2008–11 to 6.4% and 4.5% in 2016–19, respectively (table 2). In 2020, this decline persisted, while hypertensive diseases and diabetes were reported more frequently as comorbidities.

**Table 2:** Percentage of dementia-related deaths with death certificates mentioning common comorbidities (MCOD) or selected as UCOD

		2008–11	2012–15	2016–19	2020
Alzheimer’s disease and other dementias	MCOD	*n* = 17 990	*n* = 20 812	*n* = 24 093	*n* = 7406
(F01, F03, G30)	UCOD	51.1	53	57.1	48.9
Ischemic heart diseases	MCOD	19.6	16.6	14.1	12.6
(I20–I25)	UCOD	9.8	8.1	6.4	6
Cerebrovascular diseases	MCOD	15	13.2	12.5	11.2
(I60–I69)	UCOD	5.6	4.8	4.5	3.3
Hypertensive diseases	MCOD	20.8	21.4	22.1	23.6
(I10.I13)	UCOD	4.9	5.5	5.4	6.1
Diabetes	MCOD	12.6	11.8	12.2	12.6
(E10–E14)	UCOD	2.6	2.4	2	2.2
Neoplasms	MCOD	11.1	10.8	10.4	10
(C00–D48)	UCOD	5.7	5.2	5	4.7
COPD	MCOD	7	5.7	5.1	4.6
(J40–J44, J47)	UCOD	2.2	1.8	1.5	1.3
COVID-19	MCOD	–	–	–	14.5
(U07.1, U07.2)	UCOD	–	–	–	11.8

Note: UCOD, underlying cause of death; MCOD, multiple causes of death; COPD, chronic obstructive pulmonary disease.

**Figure 2:**
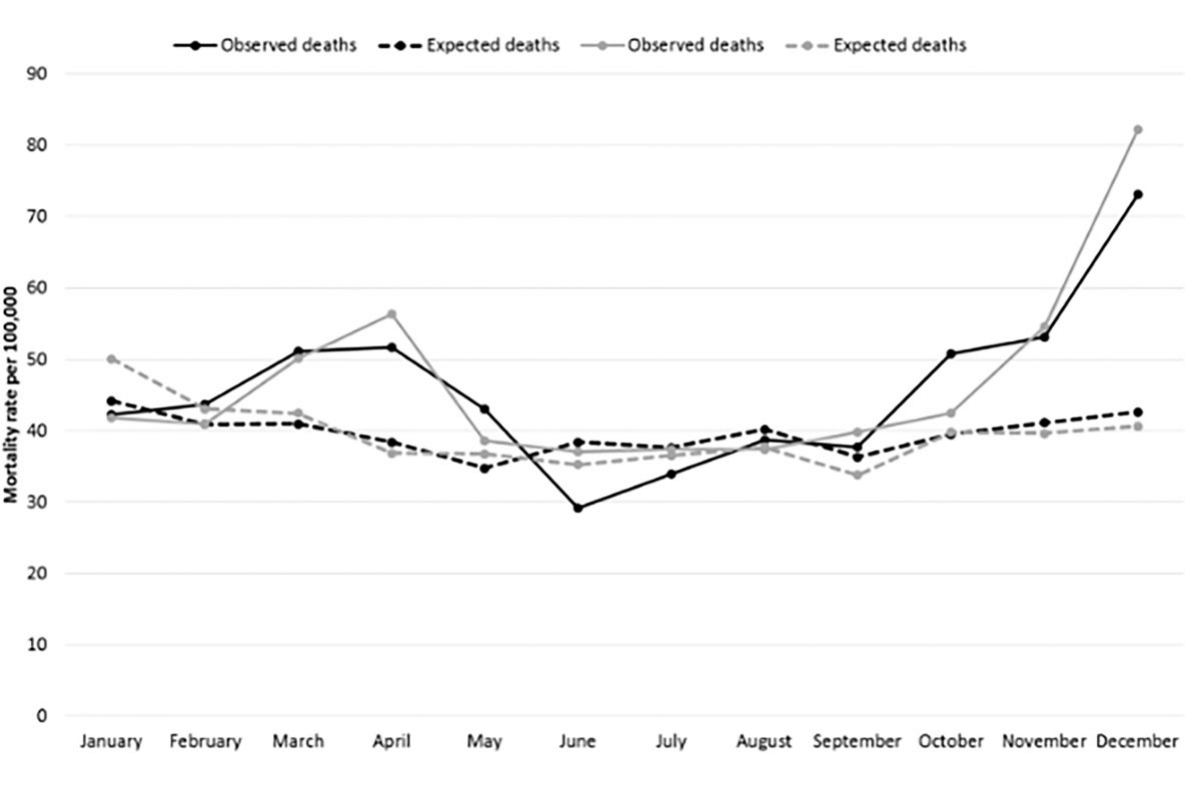
Comparing dementia-related age-standardized* monthly mortality rates (2020; MCOD) with expected rates (2008–19 time series) by sex. MCOD, multiple causes of death. *Reference: European population 2013

The monthly age-standardized dementia-related mortality rates for 2020 (MCOD) were stratified by sex (figure 2), together with the expected rates obtained from the SARIMA model applied to the 2008–19 data (Supplementary table b). Notably, an excess mortality rate was observed in 2020: a 15.5% increase in males (548.3/100 000 [95% CI 526.1–571.1] vs. an expected rate of 474.7/100 000) and an 18.3% increase in females (558.5/100 000 [95% CI 542.8–574.5] vs. an expected rate of 472.2/100 000). This increase was pronounced during the first and second pandemic waves, with both sexes showing a similar pattern.

The place of death was analyzed from 2012 and revealed that most individuals died in hospitals (41%), followed by nursing homes (35%) and homes (20%) (table 1). The number of deaths that occurred at home remained approximately stable, while those occurring in hospitals decreased and those in nursing homes progressively increased. Compared with the 2018–19 average, the greatest increase in deaths in 2020 was observed in nursing homes (+32%), followed by at home (+26%) and in hospitals (+12%) (Supplementary figure b).

## Discussion

During the early period of the COVID-19 pandemic, the vulnerable and underserved populations, including patients with ADRD, had a greater risk of dying from COVID-19. An elevated mortality rate for COVID-19 among older people is well-documented,^14–18^ and there exists evidence suggesting that ADRD may also be an age-independent risk factor for COVID-19 severity.^19^^,^^20^ In the overall population, COVID-19 infections were estimated to be responsible for approximately 83% of the observed excess mortality in 2020.^5^ Nevertheless, the overall mortality increase also reflected the indirect consequences of the pandemic, such as reduced access to healthcare services, which particularly affected patients with ADRD. In the early phase of the COVID-19 pandemic, factors such as older age, impaired autonomy and residing in nursing homes contributed to an especially high excess mortality in people with ADRD.^5^^,^^21^

The results of our study confirm excess dementia-related mortality in 2020, with an increase of 15.5% and 18.3% in males and females, respectively, in comparison with the expected figures obtained via the time series analysis. At the peak of the pandemic in the Veneto Region, in December 2020, the increase in dementia-related mortality in females exceeded 100%.

Regarding the place of death, nursing homes showed the greatest increase compared with the 2018–19 average (+32%), followed by deaths at home (+26%) and in hospitals (+12%). Nursing homes house a high proportion of patients with age-related frailty and vulnerability as well as cognitive impairment, thereby increasing this population’s susceptibility to COVID-19. Individuals with dementia are less likely to recognize and report symptoms of the infection and comply with safety recommendations (e.g. regularly washing hands, using masks and performing physical distancing), further increasing their risk of SARS-CoV-2 infection and mortality. Atypical and misleading manifestations of COVID-19, limited access to intensive care and supportive therapies, and spread of the virus, particularly in nursing homes, lead to increased mortality in the ADRD population.^22^ A previous study on the risk of infection, hospitalization and mortality due to COVID-19 in nursing home residents, compared to matched non-nursing home counterparts has shown how people in this setting were at a significantly increased risk of any of the three outcomes, especially in the first and second pandemic waves.^23^

When comparing the 2020 monthly mortality rates with the predicted estimates for the same period using the SARIMA model, peaks in excess mortality could be observed during the first (March–April) and second (October–December) pandemic waves. Despite the strong heterogeneity in the spread of COVID-19 across nations, the second pandemic wave had a much more damaging effect in several European countries, including Italy and the Veneto Region, compared with the first wave.^24^ In fact, the impact of the first pandemic wave was strongly reduced by the imposition of a lockdown that along with other public health measures, effectively contrasted the circulation of COVID-19.^25^ On the other hand, during the second wave, increased individual mobility and contacts between people led to a greater and uncontrolled spread of the virus. Furthermore, the presence of new variants characterized by high contagiousness also winded up making public health measures against the diffusion of the virus less effective.^26^ This resulted in a relatively low mortality peak during the first wave (comparable to that observed during the flu season), and a very high mortality during the second wave, confirming the much higher pathogenicity of COVID-19 compared to seasonal flu. Only after the implementation of the vaccination campaign, starting from nursing home residents and personnel, the impact of subsequent pandemic waves was reduced, at least in terms of mortality.^23^

Another study examining diabetes as MCOD also highlighted the especially steep mortality increase in diabetes during the second pandemic wave.^27^ Similarly, Parkinson’s disease-related mortality also mimicked the COVID-19 pandemic waves.^28^ The timing and intensity of the COVID-19 waves varied between the different countries and regions, and sparse data, limited to the UCOD, were available regarding ADRD-related mortality from the pandemic’s first year. In Australia, wherein no major COVID-19 pandemic wave was registered in 2020, ADRD-related deaths decreased by 5% compared with the expected numbers obtained from the 2015–19 time series.^29^ In the USA, the excess in age-standardized mortality rates for AD in 2020 was 8.7% more compared with 2019 with differences across ethnic groups.^30^ Other studies reporting an excess in ADRD-related deaths did not include all data from 2020 and were limited to the first pandemic wave; Grande et al.^7^ reported a +13.9% excess (March–April) in Italy and Wu et al.^8^ reported an excess of +28% (March–June) in England and Wales. The numbers are likely higher because the proportion of excess deaths directly associated with COVID-19 has been largely underestimated because of the frequent underreporting of SARS-CoV-2 infections.

Hence, to the best of our knowledge, this is the first study to report ADRD-related mortality during the different COVID-19 pandemic waves in 2020 and compare the standard mortality statistics based on the UCOD vs. MCOD approaches.

Different methodological approaches, such as UCOD and MCOD, can yield different results for cause-specific mortality, which is important when analyzing pandemic mortality data. In 2020, Adair et al.^11^ highlighted the added value of the MCOD approach as a more appropriate tool to examine dementia-related mortality trends. In the present study, the proportion of deaths with dementia as the UCOD increased before the pandemic (2008–19), whereas the number of deaths from other causes showed a decrease. This can be attributed to the reduction in the number of deaths caused by diseases such as ischemic heart or cerebrovascular disease. This trend showed a change in 2020 when the proportion of deaths from dementia (UCOD) decreased by 8.2%, which can be best explained by deaths being attributed to COVID-19 (11.8%). Notably, an increase in the mention of dementia in death certificates that also included diabetes or hypertensive diseases, both of which are risk factors of severe COVID-19, was observed in 2020.^31^ Only using the MCOD approach enabled the observation of an increase in age-standardized dementia-related mortality rates in 2020, clearly demonstrating the enhanced accuracy of the MCOD approach compared with the UCOD approach in providing valuable insights into both the long-term trends of dementia-related mortality and peaks associated with the pandemic waves. Furthermore, the MCOD approach most likely lowers the risk of bias due to changes in coding rules for identifying the UCOD, as well as by eliminating the competing causes of death, such as COVID-19, that are more likely to be selected as the UCOD.

The relatively long observation period (2008–20) of the current study allowed for the long-term examination of changes in dementia-related mortality and increased robustness of the expected estimates of the same compared with those observed in 2020. Furthermore, analyzing the most common comorbidities among patients with dementia provided a more complete clinical overview of the long-term variations of the causes of mortality and concomitant conditions reported in the death certificates. The reporting of hypertensive diseases and diabetes among dementia decedents was noted to increase during the pandemic, suggesting that patients with dementia having these comorbidities might be especially vulnerable to COVID-19.

The analysis of the monthly variations of the place of death provides a better understanding of the most critical environments for patients with dementia. The results of these analyses highlight where excess deaths might occur the most, thereby informing policymakers regarding environments in need of extensive attention and allocation of resources for appropriate patient care in a similar pandemic context.

There are some limitations to this study. First, dementia is often underreported in death certificates of people with a clinical diagnosis; hence, despite using the MCOD approach, there remains a possibility of underestimating the overall mortality burden.^32^ Second, only the deaths up to December 2020 were included in the study, as consolidated mortality data for 2021 were not available at the time of the analyses. Although extending the study period would have allowed for the examination of the impact of the COVID-19 pandemic for a longer period, the presence of numerous confounding factors, including the effects of mass vaccination and previous infection, would have further complicated data interpretation. A possible advantage of focusing on the first two pandemic waves included studying a naïve population that was entirely vulnerable to SARS-CoV-2, especially during the first wave. Another limitation is the possible underdiagnosis of SARS-CoV-2 infections, particularly at the beginning of the pandemic, owing to the dearth of diagnostic test availability, which could have underestimated the COVID-19-related mortality rate.

In conclusion, the analysis with the MCOD approach showed increased age-standardized dementia-related mortality rate in 2020, while the UCOD approach did not show any change, supporting the validity and wider application of the MCOD-based mortality analyses. Disease prevention strategies should consider that people with ADRD, especially those living in nursing homes, require additional attention. Promotion of COVID-19 booster doses for both people at risk and their caregivers, avoiding crowded places, wearing personal protective equipment in high-risk settings (e.g. nursing homes and healthcare facilities), frequent hand washing, immediate testing in case of symptoms and close contacts, and timely isolation measures of positive cases are likely to be among the most effective measures to contrast the spread of COVID-19, in the current context where most of the population has already been vaccinated. Future studies with data spanning the entire period of the COVID-19 pandemic are warranted to provide further evidence on the burden of dementia-related mortality across the multiple pandemic waves before and after the mass COVID-19 vaccination campaign.

## Supplementary Material

ckad005_Supplementary_Data

## Supplementary data

Key points

- The COVID-19 pandemic impacted long-term dementia-related mortality trends.
- Dementia-related mortality rates showed an increase in 2020 compared with the pre-pandemic period when determined using the multiple causes of death (MCOD) approach but not when applying the underlying cause of death (UCOD) approach.
- Nursing homes were the most critical setting for dementia patients, with a 32% increase in deaths during 2020 compared with the 2018–19 average.
- MCOD proved to be more robust than the usually applied UCOD, and hence, should be included in future analyses.
