# Supplementary material for: Veneto Region dementia-related mortality during the COVID-19 pandemic: multiple causes of death and time series analysis
Source: Eur J Public Health. 2023 Feb 27;33(2):190–5. doi: 10.1093/eurpub/ckad005 (PMC10066484; doi:10.1093/eurpub/ckad005)
Supplement: ckad005_Supplementary_Data [file ckad005_supplementary_data.docx]

**Supplementary Data**

**Figure a.** Age-specific dementia-related mortality rates (per 100,000 inhabitants) stratified by sex.

| **Age class (years)** | **Men** | **Women** |
| --- | --- | --- |
| **65**–**69** | 20.9 | 15.3 |
| **70**–**74** | 72.1 | 51.9 |
| **75**–**79** | 234.3 | 189.7 |
| **80**–**84** | 701.5 | 601.0 |
| **85**–**89** | 1,707.4 | 1,647.4 |
| **90+** | 3,587.1 | 4,163.1 |

**Table a**. Dementia-related age-standardized* mortality rates (per 100,000 inhabitants) from 2008 to 2020 for both underlying (UCOD) and multiple causes (MCOD) of death, 95% confidence interval (95% CI) and percent variation compared to the previous year: by sex and calendar year.

| Sex |  | UCOD | | | MCOD | | |
| --- | --- | --- | --- | --- | --- | --- | --- |
|  | **Year** | **Rate x 100,000** | **(95% CI)** | **Percent variation** | **Rate x 100,000** | **(95% CI)** | **Percent variation** |
| Male | 2008 | 218.1 | (200.1-237.3) | - | 465.6 | (438.9-493.4) | - |
|  | 2009 | 266.0 | (245.8-287.3) | 21.95 | 492.0 | (464.6-520.4) | 5.65 |
|  | 2010 | 243.4 | (224.0-264.0) | -8.49 | 488.7 | (461.1-517.5) | -0.66 |
|  | 2011 | 251.0 | (232.1-271.0) | 3.13 | 487.9 | (461.6-515.2) | -0.17 |
|  | 2012 | 242.8 | (225.1-261.5) | -3.26 | 474.5 | (449.7-500.2) | -2.74 |
|  | 2013 | 250.8 | (233.3-269.2) | 3.26 | 457.3 | (433.7-481.7) | -3.63 |
|  | 2014 | 244.9 | (228.0-262.8) | -2.32 | 460.1 | (437.0-484.2) | 0.63 |
|  | 2015 | 246.3 | (229.9-263.6) | 0.56 | 480.8 | (457.7-504.6) | 4.48 |
|  | 2016 | 257.2 | (240.9-274.3) | 4.43 | 482.3 | (459.8-505.6) | 0.32 |
|  | 2017 | 254.3 | (238.4-270.9) | -1.15 | 475.2 | (453.3-497.8) | -1.47 |
|  | 2018 | 279.5 | (263.0-296.9) | 9.94 | 500.8 | (478.5-523.7) | 5.39 |
|  | 2019 | 273.7 | (257.7-290.4) | -2.09 | 458.5 | (437.8-480.0) | -8.43 |
|  | **2020** | **256.1** | **(240.9-271.9)** | **-6.44** | **548.3** | **(526.1-571.1)** | **19.6** |
| Female | 2008 | 239.0 | (227.0-251.5) | - | 466.5 | (449.7-483.8) | - |
|  | 2009 | 242.4 | (230.3-255.0) | 1.40 | 456.7 | (440.1-473.9) | -2.09 |
|  | 2010 | 244.9 | (232.9-257.5) | 1.06 | 489.1 | (472.0-506.8) | 7.10 |
|  | 2011 | 239.7 | (228.2-251.7) | -2.13 | 461.1 | (445.0-477.6) | -5.74 |
|  | 2012 | 253.3 | (241.7-265.3) | 5.65 | 481.9 | (465.9-498.4) | 4.51 |
|  | 2013 | 238.4 | (227.3-249.9) | -5.88 | 447.1 | (432.0-462.7) | -7.21 |
|  | 2014 | 239.2 | (228.3-250.4) | 0.34 | 440.5 | (425.7-455.7) | -1.49 |
|  | 2015 | 269.9 | (258.6-281.7) | 12.85 | 497.7 | (482.3-513.5) | 12.98 |
|  | 2016 | 272.5 | (261.2-284.2) | 0.95 | 495.0 | (479.7-510.7) | -0.54 |
|  | 2017 | 282.4 | (271.0-294.2) | 3.64 | 488.8 | (473.8-504.2) | -1.26 |
|  | 2018 | 282.1 | (270.8-293.7) | -0.12 | 487.0 | (472.2-502.2) | -0.36 |
|  | 2019 | 277.4 | (266.3-288.8) | -1.66 | 460.2 | (445.9-474.9) | -5.50 |
|  | **2020** | **281.0** | **(269.9-292.4)** | **1.30** | **558.5** | **(542.8-574.5)** | **21.35** |

*Reference: European population 2013

**Table b.** Dementia-related age-standardized* monthly mortality rates (per 100,000 inhabitants) (MCOD) observed in 2020 compared to expected based on the 2008–2019 time-series and percentage excess by sex. Veneto Region, Italy.

| Month | Male | | | Female | | |
| --- | --- | --- | --- | --- | --- | --- |
|  | Observed | Expected | Percentage excess | Observed | Expected | Percentage excess |
| January | 42.21 | 44.17 | -4.4 | 41.74 | 50.03 | -16.6 |
| February | 43.65 | 40.87 | 6.8 | 40.91 | 43.05 | -5.0 |
| March | 51.12 | 40.95 | 24.8 | 50.18 | 42.46 | 18.2 |
| April | 51.72 | 38.40 | 34.7 | 56.30 | 36.84 | 52.8 |
| May | 43.03 | 34.72 | 24.0 | 38.59 | 36.70 | 5.2 |
| June | 29.18 | 38.40 | -24.0 | 37.05 | 35.23 | 5.2 |
| July | 33.86 | 37.58 | -9.9 | 37.32 | 36.50 | 2.2 |
| August | 38.71 | 40.17 | -3.6 | 37.34 | 37.66 | -0.9 |
| September | 37.71 | 36.30 | 3.9 | 39.81 | 33.78 | 17.8 |
| October | 50.82 | 39.47 | 28.8 | 42.49 | 39.78 | 6.8 |
| November | 53.14 | 41.10 | 29.3 | 54.58 | 39.61 | 37.8 |
| December | 73.10 | 42.59 | 71.6 | 82.16 | 40.58 | 102.5 |

*Reference: European population 2013

**Figure b.** Dementia-related mortality variation in 2020 compared to the 2018–2019 average (100%) stratified by place of death.
